# Comment on: Surgical treatment of anorectal melanoma: a systematic review and meta-analysis

**DOI:** 10.1093/bjsopen/zrac015

**Published:** 2022-02-09

**Authors:** Mufaddal Kazi, Ambarish Chatterjee, Avanish Saklani

**Affiliations:** Department of Colorectal Surgical Oncology, Tata Memorial Centre, Homi Bhabha National Institute (HBNI), Mumbai, Maharashtra 400012, India

*Dear Editor*

In the aggregate-data meta-analysis of operated anorectal melanomas, Jutten et al. found no difference in survival between local excision or radical resection regardless of stage^1^. We find the conclusions to be overstated and incautious given the high risk of biases that were not adequately recognized.

The inherent selection of small tumours without sphincter involvement for less extensive resection against larger, deep tumours for radical resection exists and cannot be eliminated by meta-analysis of included studies. Thus, equivalent survivals for higher risk tumours with radical resection compared to local excision for low-risk melanomas supports the effectiveness of more extensive resection in regional disease. Without correcting for tumour size, thickness, depth of invasion, KIT and BRAF mutations, and PD-L1 expression, matching similar staged cohorts is insufficient from summary statistics.

In the ‘risk of confounding bias’ domain, 11/34 studies (32.3 per cent) had serious risks while 9/34 (26.4 per cent) had low risk in the ROBINS-I tool. Assigning a ‘low risk’ implies that similarities in the two intervention arms were akin to a randomized study. This is not possible as the studies with largest weights in the meta-analysis were from population databases with serious risk of confounding and missing data. Further, no direction in the risk of bias was provided for any domains.

Most included studies spanned over decades without separation by the year of treatment or individual participant data. Therefore, it is unclear how the time-interval stratification was done for analysis. Further, when looking at differences in outcomes based on the continent of origin, a SEER database used by authors from China was considered Asian data. Finally, the authors have used odds-ratio as a summary statistic for time-to-event outcomes. Odds-ratio is inappropriate as not all patients had events, and follow up duration of studies and individual patients were non-homogenous.

The study results have the most decisive implications for node-positive melanomas where local excision appears to be justified by the conclusions. However, as only 111 (6 per cent) had stages I and II separated where stratified results were available, interpretation in this subset should be guarded. In addition, the proportion of positive margins, local recurrences and completion radical resections required after local excision is vital to decide on the surgery offered. Thus, local control and quality of life outcomes are vital for future studies to report.

## Data Sharing Statement

No new data was generated for the present correspondence

*Disclosure*. The authors declare no conflict of interest.
